# 3-(2,4-Di­chloro­phen­oxy)-1-(4-meth­oxy­benz­yl)-4-(4-nitro­phen­yl)azetidin-2-one

**DOI:** 10.1107/S1600536814015013

**Published:** 2014-07-02

**Authors:** Zeliha Atioğlu, Mehmet Akkurt, Aliasghar Jarrahpour, Roghayeh Heiran, Namık Özdemir

**Affiliations:** a Ilke Education and Health Foundation, Cappadocia Vocational College, The Medical Imaging Techniques Program, 50420 Mustafapaşa, Ürgüp, Nevşehir, Turkey; b Department of Physics, Faculty of Sciences, Erciyes University, 38039 Kayseri, Turkey; c Department of Chemistry, College of Sciences, Shiraz University, 71454 Shiraz, Iran; d Department of Physics, Faculty of Arts and Sciences, Ondokuz Mayıs University, 55139 Samsun, Turkey

**Keywords:** crystal structure

## Abstract

The β-lactam ring of the title compound, C_23_H_18_Cl_2_N_2_O_5_, is nearly planar [maximum deviation = 0.019 (2) Å for the N atom] and its mean plane makes dihedral angles of 56.86 (15), 68.83 (15) and 83.75 (15)° with the di­chloro-, nitro- and meth­oxy-substituted benzene rings, respectively. In the crystal, mol­ecules are linked by pairs of C—H⋯O hydrogen bonds, forming inversion dimers with *R*_2_^2^(10) loops. The dimers are linked by further C—H⋯O hydrogen bonds, forming sheets lying parallel to (001). The mol­ecular packing is further stabilized by C—H⋯π inter­actions.

## Related literature   

For general background to β-lactams, see: Schunk & Enders (2000 ▶); France *et al.* (2004 ▶); Pitts & Lectka (2014 ▶); Arya *et al.* (2014 ▶); Banik *et al.* (2003 ▶); Delpiccolo *et al.* (2003 ▶); Hodous & Fu (2002 ▶). For the crystal structures of some β-lactams, see: Akkurt *et al.* (2011 ▶); Butcher *et al.* (2011 ▶). 
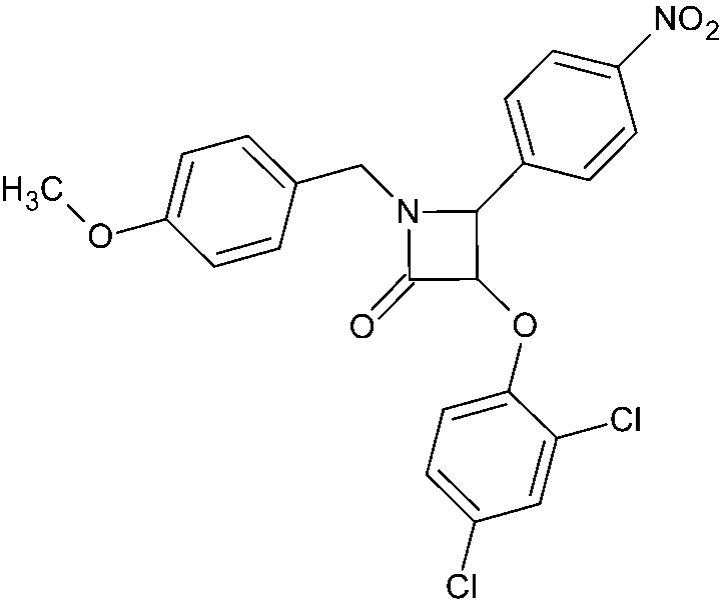


## Experimental   

### 

#### Crystal data  

C_23_H_18_Cl_2_N_2_O_5_
*M* *_r_* = 473.29
Monoclinic, 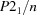

*a* = 5.0716 (5) Å
*b* = 20.9390 (12) Å
*c* = 20.1516 (18) Å
β = 96.457 (7)°
*V* = 2126.4 (3) Å^3^
*Z* = 4
Mo *K*α radiation
μ = 0.35 mm^−1^
*T* = 296 K
0.59 × 0.28 × 0.06 mm

#### Data collection  

Stoe IPDS 2 diffractometer
Absorption correction: integration (*X-RED32*; Stoe & Cie, 2002 ▶) *T*_min_ = 0.901, *T*_max_ = 0.972
15060 measured reflections
4179 independent reflections
2123 reflections with *I* > 2σ(*I*)
*R*_int_ = 0.062

#### Refinement  

*R*[*F* ^2^ > 2σ(*F* ^2^)] = 0.042
*wR*(*F* ^2^) = 0.061
*S* = 0.85
4179 reflections
289 parameters
H-atom parameters constrained
Δρ_max_ = 0.16 e Å^−3^
Δρ_min_ = −0.19 e Å^−3^

### 

Data collection: *X-AREA* (Stoe & Cie, 2002 ▶); cell refinement: *X-AREA*; data reduction: *X-RED32* (Stoe & Cie, 2002 ▶); program(s) used to solve structure: *SHELXS2013* (Sheldrick, 2008 ▶); program(s) used to refine structure: *SHELXL2013* (Sheldrick, 2008 ▶); molecular graphics: *ORTEP-3 for Windows* (Farrugia, 2012 ▶); software used to prepare material for publication: *WinGX* (Farrugia, 2012 ▶).

## Supplementary Material

Crystal structure: contains datablock(s) global, I. DOI: 10.1107/S1600536814015013/su2747sup1.cif

Structure factors: contains datablock(s) I. DOI: 10.1107/S1600536814015013/su2747Isup2.hkl

Supporting information file. DOI: 10.1107/S1600536814015013/su2747Isup3.cml

CCDC reference: 1010327

Additional supporting information:  crystallographic information; 3D view; checkCIF report

## Figures and Tables

**Table 1:** Hydrogen-bond geometry (Å, °) *Cg* is the centroid of the C17–C22 benzene ring.

*D*—H⋯*A*	*D*—H	H⋯*A*	*D*⋯*A*	*D*—H⋯*A*
C3—H3⋯O1^i^	0.98	2.58	3.417 (3)	143
C6—H6⋯O5^ii^	0.93	2.57	3.328 (3)	139
C12—H12⋯O3^iii^	0.93	2.57	3.495 (4)	176
C16—H16*A*⋯*Cg* ^iv^	0.97	2.70	3.649 (3)	166

Symmetry codes: (i) 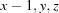; (ii) 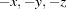; (iii) 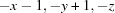; (iv) 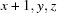.

**Fig. 1.**
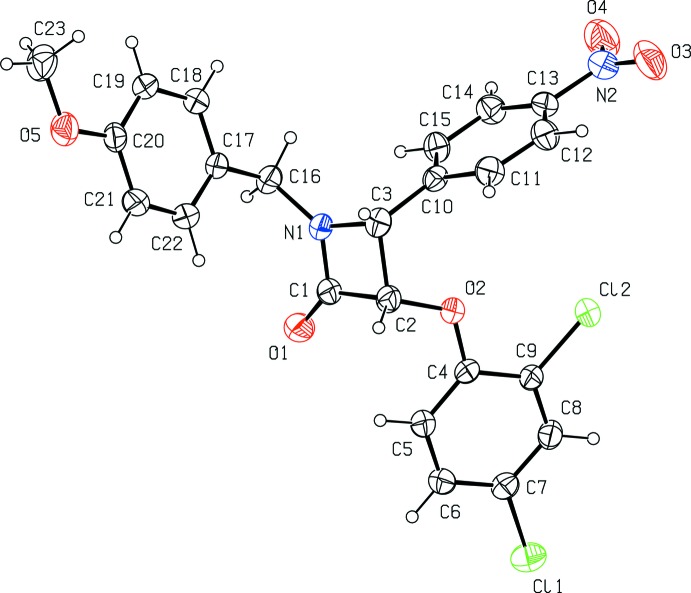
The molecular structure of the title molecule, with atom labelling. Displacement ellipsoids are drawn at the 30% probability level.

**Fig. 2.**
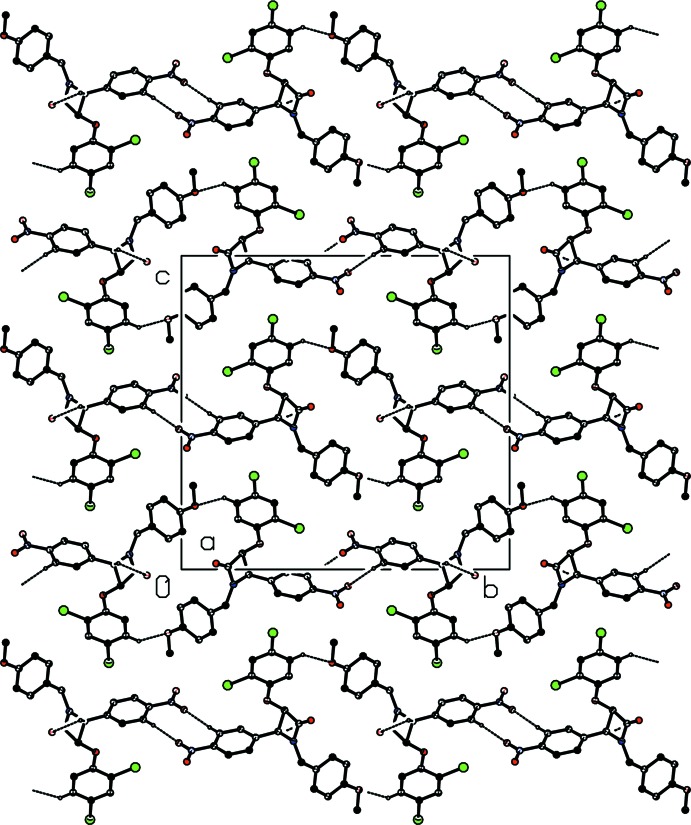
A view along the a axis of the crystal packing of the title compound. Hydrogen bonds are shown as dashed lines (see Table 1 for details).

## supplementary crystallographic information

### S1. Comment

The β-lactam (2-azetidinone) ring is the most well known heterocycle to have been studied during the last century (Pitts & Lectka, 2014; France *et al.*, 2004; Arya *et al.*, 2014). The β-lactam framework is the structural element of a large class of broad-spectrum antibiotics such as penicillins, cephalosporins and monobactams (Delpiccolo *et al.*, 2003; Schunk & Enders, 2000; Banik *et al.*, 2003), that effectively combat bacterial infections (Schunk & Enders, 2000). However, the need for new antibiotics has been growing, as a result of the rapid emergence of bacterial strains' resistance to traditional drugs (Hodous & Fu, 2002; Delpiccolo *et al.*, 2003). Therefore, in continuation of our research on the synthesis of β-lactams, we describe herein the synthesis and crystal structure of the title compound.

In the title molecule, Fig. 1, the β-lactam ring (N1/C1–C3) is nearly planar with a maximum deviation of -0.016 (1) Å for atom N1. The mean plane of this four-membered β-lactam ring is twisted from the planes of the dichloro-, nitro- and methoxy substituted benzene rings, making the dihedral angles of 56.86 (15), 68.83 (15) and 83.75 (15)°, respectively. The bond lengths and bond angles are within normal values and are comparable with those reported for similar compounds (Akkurt *et al.*, 2011; Butcher *et al.*, 2011).

In the crystal, molecules are linked by a pair of C—H···O hydrogen bonds forming inversion dimers with R^2^_2_(10) loops (Table 1 and Fig. 2). The dimers are linked by further C-H···O hydrogen bonds forming sheets lying parallel to (001). The molecular packing is further stabilized by C—H···π interactions (Table 1).

### S2. Experimental

A mixture of *N*-(4-nitrobenzylidene) (4-methoxyphenyl) methanamine (0.27 g, 1.00 mmol), 2,4-dichlorophenoxyacetic acid (0.34 g, 1.50 mmol), tosyl chloride (0.28 g, 1.50 mmol) and triethylamine (0.25 g, 2.50 mmol) in dry CH_2_Cl_2_ was stirred at room temperature overnight. After completion of the reaction, monitored by TLC, the mixture was washed with HCl (1 N), saturated sodium bicarbonate solution, brine, dried over anhydrous Na_2_SO_4_ and the solvent was then evaporated under vacuum to afford the crude product. This was purified by recrystallization from EtOAc giving pale yellow prismatic crystals on slow evaporation of the solvent (yield 72%). M.p. 397 - 399 K. Spectroscopic data for the title compound are given in the archived CIF.

### S3. Refinement

All the H atoms were positioned geometrically and refined using a riding model: C—H = 0.93 - 0.98 Å with *U*_iso_(H) = 1.2*U*_eq_(C).

### Crystal data

**Table:**

C_23_H_18_Cl_2_N_2_O_5_	*F*(000) = 976
*M**_r_* = 473.29	*D*_x_ = 1.478 Mg m^−^^3^
Monoclinic, *P*2_1_/*n*	Mo *K*α radiation, λ = 0.71073 Å
Hall symbol: -P 2yn	Cell parameters from 10788 reflections
*a* = 5.0716 (5) Å	θ = 1.4–28.4°
*b* = 20.9390 (12) Å	µ = 0.35 mm^−^^1^
*c* = 20.1516 (18) Å	*T* = 296 K
β = 96.457 (7)°	Prism, pale yellow
*V* = 2126.4 (3) Å^3^	0.59 × 0.28 × 0.06 mm
*Z* = 4	

### Data collection

**Table:**

Stoe IPDS 2 diffractometer	4179 independent reflections
Radiation source: sealed X-ray tube, 12 x 0.4 mm long-fine focus	2123 reflections with *I* > 2σ(*I*)
Plane graphite monochromator	*R*_int_ = 0.062
Detector resolution: 6.67 pixels mm^-1^	θ_max_ = 26.0°, θ_min_ = 1.4°
ω scans	*h* = −6→6
Absorption correction: integration (*X-RED32*; Stoe & Cie, 2002)	*k* = −25→25
*T*_min_ = 0.901, *T*_max_ = 0.972	*l* = −24→24
15060 measured reflections	

### Refinement

**Table:**

Refinement on *F*^2^	0 restraints
Least-squares matrix: full	Hydrogen site location: inferred from neighbouring sites
*R*[*F*^2^ > 2σ(*F*^2^)] = 0.042	H-atom parameters constrained
*wR*(*F*^2^) = 0.061	*w* = 1/[σ^2^(*F*_o_^2^) + (0.0162*P*)^2^] where *P* = (*F*_o_^2^ + 2*F*_c_^2^)/3
*S* = 0.85	(Δ/σ)_max_ < 0.001
4179 reflections	Δρ_max_ = 0.16 e Å^−^^3^
289 parameters	Δρ_min_ = −0.19 e Å^−^^3^

### Special details

**Table:**

Experimental. Spectroscopic data for the title compound:IR (KBr, cm^-1^): 1758 (CO β-lactam), 1352, 1520 (NO_2_). ^1^H-NMR (CDCl_3_) δ (p.p.m.): 3.78 (OMe, s, 3H), 3.94 (CH_2_, d, J =14.6 Hz, 1H), 4.78 (CH_2_, d, J = 14.6 Hz, 1H), 4.85 (H-4, d, J = 4.8 Hz, 1H), 5.38 (H-3, d, J = 4.8 Hz, 1H), 6.80 (ArH, d, J = 8.7 Hz, 2H), 7.00 (ArH, d, J = 8.8 Hz, 1H), 7.03 (ArH, d, J = 8.7 Hz, 2H), 7.08 (ArH, d, J = 8.8 Hz, 1H), 7.19 (ArH, s, 1H), 7.45 (ArH, d, J = 8.8 Hz, 2H), 8.17 (ArH, d, J = 8.8 Hz, 2H). ^13^C-NMR (CDCl_3_) δ (p.p.m.): 44.4 (CH_2_), 55.3 (OMe) 59.8 (C-4), 82.5 (C-3), 114.3, 116.2, 123.4, 123.8, 125.8, 127.5, 127.7, 129.4, 130.0, 130.1, 140.3, 148.1, 151.1, 159.5 (aromatic carbon), 164.36 (CO β-lactam). MS m/*z* = 472 [*M*^+^].
Geometry. Bond distances, angles *etc*. have been calculated using the rounded fractional coordinates. All su's are estimated from the variances of the (full) variance-covariance matrix. The cell e.s.d.'s are taken into account in the estimation of distances, angles and torsion angles
Refinement. Refinement on *F*^2^ for ALL reflections except those flagged by the user for potential systematic errors. Weighted *R*-factors *wR* and all goodnesses of fit *S* are based on *F*^2^, conventional *R*-factors *R* are based on *F*, with *F* set to zero for negative *F*^2^. The observed criterion of *F*^2^ > σ(*F*^2^) is used only for calculating -*R*-factor-obs *etc*. and is not relevant to the choice of reflections for refinement. *R*-factors based on *F*^2^ are statistically about twice as large as those based on *F*, and *R*-factors based on ALL data will be even larger.

### Fractional atomic coordinates and isotropic or equivalent isotropic displacement parameters (Å^2^)

**Table:**

	*x*	*y*	*z*	*U*_iso_*/*U*_eq_	
Cl1	0.91503 (14)	0.22124 (4)	0.29769 (3)	0.0722 (3)	
Cl2	0.23669 (16)	0.35928 (3)	0.13099 (3)	0.0705 (3)	
O1	0.2489 (4)	0.10566 (8)	0.01887 (8)	0.0716 (7)	
O2	0.0396 (3)	0.23885 (7)	0.08223 (8)	0.0598 (6)	
O3	−0.2578 (5)	0.50783 (10)	−0.05297 (12)	0.1058 (10)	
O4	0.0490 (5)	0.48354 (10)	−0.11293 (12)	0.1037 (10)	
O5	−0.9069 (4)	−0.04073 (8)	−0.20251 (9)	0.0712 (7)	
N1	−0.0881 (4)	0.15859 (8)	−0.04739 (9)	0.0501 (7)	
N2	−0.1189 (6)	0.46924 (12)	−0.07734 (13)	0.0730 (11)	
C1	0.0631 (5)	0.14198 (12)	0.00884 (11)	0.0488 (9)	
C2	−0.0932 (5)	0.18554 (11)	0.05155 (11)	0.0499 (9)	
C3	−0.2519 (5)	0.20533 (11)	−0.01636 (11)	0.0493 (8)	
C4	0.2330 (5)	0.23048 (11)	0.13478 (11)	0.0484 (9)	
C5	0.3203 (5)	0.17250 (12)	0.16032 (11)	0.0560 (9)	
C6	0.5282 (5)	0.16962 (12)	0.21112 (11)	0.0559 (10)	
C7	0.6458 (5)	0.22470 (13)	0.23584 (11)	0.0518 (9)	
C8	0.5564 (5)	0.28303 (12)	0.21190 (11)	0.0561 (9)	
C9	0.3495 (5)	0.28593 (11)	0.16161 (10)	0.0493 (9)	
C10	−0.2222 (4)	0.27389 (11)	−0.03557 (10)	0.0438 (8)	
C11	−0.3762 (5)	0.31962 (12)	−0.00929 (12)	0.0576 (10)	
C12	−0.3445 (6)	0.38355 (13)	−0.02211 (13)	0.0638 (11)	
C13	−0.1588 (6)	0.40110 (12)	−0.06275 (12)	0.0536 (10)	
C14	−0.0052 (5)	0.35691 (13)	−0.09064 (12)	0.0588 (10)	
C15	−0.0372 (5)	0.29338 (12)	−0.07617 (11)	0.0547 (9)	
C16	−0.0924 (5)	0.13434 (12)	−0.11484 (10)	0.0558 (9)	
C17	−0.3177 (5)	0.08960 (11)	−0.13711 (11)	0.0465 (9)	
C18	−0.4539 (5)	0.09594 (11)	−0.19956 (11)	0.0526 (9)	
C19	−0.6509 (5)	0.05349 (12)	−0.22383 (11)	0.0540 (9)	
C20	−0.7172 (5)	0.00404 (12)	−0.18411 (13)	0.0529 (9)	
C21	−0.5827 (6)	−0.00258 (12)	−0.12111 (13)	0.0647 (10)	
C22	−0.3856 (5)	0.03948 (12)	−0.09822 (12)	0.0614 (10)	
C23	−1.0492 (6)	−0.03553 (13)	−0.26693 (14)	0.0812 (12)	
H2	−0.20000	0.16210	0.08110	0.0600*	
H3	−0.43840	0.19230	−0.01930	0.0590*	
H5	0.23980	0.13510	0.14350	0.0670*	
H6	0.58760	0.13030	0.22830	0.0670*	
H8	0.63500	0.32040	0.22950	0.0670*	
H11	−0.50410	0.30700	0.01770	0.0690*	
H12	−0.44720	0.41410	−0.00350	0.0770*	
H14	0.11830	0.36960	−0.11880	0.0710*	
H15	0.06820	0.26310	−0.09420	0.0660*	
H16A	0.07350	0.11230	−0.11850	0.0670*	
H16B	−0.10090	0.17030	−0.14530	0.0670*	
H18	−0.41260	0.12980	−0.22640	0.0630*	
H19	−0.73760	0.05840	−0.26670	0.0650*	
H21	−0.62600	−0.03590	−0.09380	0.0780*	
H22	−0.29650	0.03400	−0.05570	0.0740*	
H23A	−1.17560	−0.06970	−0.27350	0.1210*	
H23B	−1.14060	0.00470	−0.27080	0.1210*	
H23C	−0.92780	−0.03800	−0.30010	0.1210*	

### Atomic displacement parameters (Å^2^)

**Table:**

	*U*^11^	*U*^22^	*U*^33^	*U*^12^	*U*^13^	*U*^23^
Cl1	0.0632 (5)	0.0887 (5)	0.0622 (4)	−0.0012 (4)	−0.0041 (3)	0.0047 (4)
Cl2	0.1082 (6)	0.0417 (4)	0.0589 (4)	0.0053 (4)	−0.0028 (4)	−0.0010 (3)
O1	0.0839 (15)	0.0666 (12)	0.0620 (11)	0.0211 (12)	−0.0019 (10)	−0.0016 (10)
O2	0.0835 (13)	0.0417 (10)	0.0498 (10)	−0.0014 (9)	−0.0113 (9)	−0.0032 (8)
O3	0.136 (2)	0.0596 (14)	0.1232 (19)	0.0290 (15)	0.0202 (16)	0.0043 (13)
O4	0.138 (2)	0.0701 (15)	0.1078 (18)	−0.0071 (14)	0.0353 (16)	0.0130 (13)
O5	0.0738 (13)	0.0555 (11)	0.0812 (13)	−0.0087 (11)	−0.0044 (10)	−0.0089 (10)
N1	0.0607 (14)	0.0447 (12)	0.0437 (12)	0.0053 (11)	0.0008 (11)	−0.0081 (9)
N2	0.090 (2)	0.0609 (19)	0.0651 (17)	0.0106 (17)	−0.0044 (14)	0.0040 (14)
C1	0.0589 (19)	0.0406 (14)	0.0465 (15)	−0.0059 (14)	0.0046 (14)	0.0002 (13)
C2	0.0589 (17)	0.0437 (15)	0.0474 (14)	−0.0096 (13)	0.0075 (13)	−0.0017 (12)
C3	0.0402 (15)	0.0532 (15)	0.0548 (14)	−0.0045 (13)	0.0068 (12)	−0.0074 (13)
C4	0.0645 (17)	0.0472 (15)	0.0338 (12)	0.0010 (14)	0.0067 (12)	−0.0024 (12)
C5	0.0755 (19)	0.0424 (15)	0.0492 (15)	−0.0051 (15)	0.0025 (14)	−0.0012 (12)
C6	0.072 (2)	0.0489 (16)	0.0470 (15)	0.0060 (15)	0.0072 (14)	0.0036 (12)
C7	0.0547 (17)	0.0572 (17)	0.0439 (14)	−0.0010 (15)	0.0075 (12)	−0.0019 (13)
C8	0.0727 (18)	0.0465 (16)	0.0496 (15)	−0.0083 (15)	0.0087 (13)	−0.0078 (13)
C9	0.0691 (18)	0.0409 (15)	0.0381 (13)	−0.0018 (14)	0.0071 (13)	−0.0026 (11)
C10	0.0395 (14)	0.0495 (15)	0.0419 (13)	0.0010 (14)	0.0020 (11)	−0.0058 (12)
C11	0.0569 (18)	0.0614 (18)	0.0568 (15)	0.0101 (15)	0.0164 (13)	−0.0009 (14)
C12	0.068 (2)	0.0553 (18)	0.0683 (18)	0.0209 (15)	0.0080 (16)	−0.0036 (14)
C13	0.0605 (19)	0.0469 (17)	0.0505 (15)	0.0055 (15)	−0.0067 (14)	0.0030 (13)
C14	0.0599 (19)	0.0576 (17)	0.0602 (16)	0.0011 (16)	0.0123 (14)	0.0032 (14)
C15	0.0550 (17)	0.0492 (17)	0.0611 (15)	0.0059 (14)	0.0118 (14)	−0.0051 (13)
C16	0.0653 (18)	0.0551 (16)	0.0463 (14)	0.0029 (15)	0.0034 (12)	−0.0103 (13)
C17	0.0563 (17)	0.0410 (14)	0.0417 (14)	0.0041 (13)	0.0037 (12)	−0.0066 (12)
C18	0.0659 (18)	0.0450 (15)	0.0460 (15)	0.0013 (14)	0.0029 (13)	0.0012 (12)
C19	0.0637 (18)	0.0499 (16)	0.0455 (14)	0.0027 (14)	−0.0061 (13)	−0.0033 (13)
C20	0.0568 (17)	0.0396 (15)	0.0610 (17)	0.0043 (14)	0.0004 (14)	−0.0092 (13)
C21	0.087 (2)	0.0469 (15)	0.0570 (17)	−0.0076 (16)	−0.0059 (15)	0.0051 (13)
C22	0.082 (2)	0.0517 (16)	0.0470 (15)	0.0020 (16)	−0.0084 (14)	0.0000 (13)
C23	0.070 (2)	0.076 (2)	0.092 (2)	−0.0058 (17)	−0.0155 (17)	−0.0193 (17)

### Geometric parameters (Å, º)

**Table:**

Cl1—C7	1.744 (2)	C14—C15	1.375 (4)
Cl2—C9	1.729 (2)	C16—C17	1.506 (3)
O1—C1	1.210 (3)	C17—C18	1.372 (3)
O2—C2	1.410 (3)	C17—C22	1.377 (3)
O2—C4	1.371 (3)	C18—C19	1.385 (3)
O3—N2	1.212 (4)	C19—C20	1.374 (4)
O4—N2	1.211 (4)	C20—C21	1.379 (4)
O5—C20	1.364 (3)	C21—C22	1.373 (4)
O5—C23	1.417 (3)	C2—H2	0.9800
N1—C1	1.341 (3)	C3—H3	0.9800
N1—C3	1.468 (3)	C5—H5	0.9300
N1—C16	1.449 (3)	C6—H6	0.9300
N2—C13	1.475 (4)	C8—H8	0.9300
C1—C2	1.534 (3)	C11—H11	0.9300
C2—C3	1.564 (3)	C12—H12	0.9300
C3—C10	1.499 (3)	C14—H14	0.9300
C4—C5	1.372 (3)	C15—H15	0.9300
C4—C9	1.385 (3)	C16—H16A	0.9700
C5—C6	1.386 (3)	C16—H16B	0.9700
C6—C7	1.367 (4)	C18—H18	0.9300
C7—C8	1.371 (4)	C19—H19	0.9300
C8—C9	1.376 (3)	C21—H21	0.9300
C10—C11	1.379 (3)	C22—H22	0.9300
C10—C15	1.374 (3)	C23—H23A	0.9600
C11—C12	1.376 (4)	C23—H23B	0.9600
C12—C13	1.366 (4)	C23—H23C	0.9600
C13—C14	1.370 (4)		
			
C2—O2—C4	120.15 (17)	O5—C20—C19	124.8 (2)
C20—O5—C23	117.9 (2)	O5—C20—C21	116.2 (2)
C1—N1—C3	96.33 (18)	C19—C20—C21	119.0 (2)
C1—N1—C16	130.5 (2)	C20—C21—C22	120.7 (2)
C3—N1—C16	133.13 (19)	C17—C22—C21	121.2 (2)
O3—N2—O4	123.7 (3)	O2—C2—H2	114.00
O3—N2—C13	117.7 (3)	C1—C2—H2	113.00
O4—N2—C13	118.6 (3)	C3—C2—H2	114.00
O1—C1—N1	131.7 (2)	N1—C3—H3	112.00
O1—C1—C2	136.2 (2)	C2—C3—H3	112.00
N1—C1—C2	92.14 (19)	C10—C3—H3	112.00
O2—C2—C1	117.7 (2)	C4—C5—H5	120.00
O2—C2—C3	110.33 (18)	C6—C5—H5	120.00
C1—C2—C3	85.08 (17)	C5—C6—H6	120.00
N1—C3—C2	86.32 (17)	C7—C6—H6	120.00
N1—C3—C10	116.77 (19)	C7—C8—H8	120.00
C2—C3—C10	115.03 (19)	C9—C8—H8	120.00
O2—C4—C5	125.1 (2)	C10—C11—H11	119.00
O2—C4—C9	115.5 (2)	C12—C11—H11	119.00
C5—C4—C9	119.3 (2)	C11—C12—H12	121.00
C4—C5—C6	120.1 (2)	C13—C12—H12	121.00
C5—C6—C7	119.8 (2)	C13—C14—H14	121.00
Cl1—C7—C6	120.0 (2)	C15—C14—H14	121.00
Cl1—C7—C8	119.3 (2)	C10—C15—H15	119.00
C6—C7—C8	120.7 (2)	C14—C15—H15	119.00
C7—C8—C9	119.5 (2)	N1—C16—H16A	108.00
Cl2—C9—C4	119.74 (18)	N1—C16—H16B	108.00
Cl2—C9—C8	119.78 (18)	C17—C16—H16A	108.00
C4—C9—C8	120.5 (2)	C17—C16—H16B	108.00
C3—C10—C11	119.2 (2)	H16A—C16—H16B	107.00
C3—C10—C15	122.2 (2)	C17—C18—H18	119.00
C11—C10—C15	118.5 (2)	C19—C18—H18	119.00
C10—C11—C12	121.4 (2)	C18—C19—H19	120.00
C11—C12—C13	118.5 (3)	C20—C19—H19	120.00
N2—C13—C12	119.9 (2)	C20—C21—H21	120.00
N2—C13—C14	118.3 (2)	C22—C21—H21	120.00
C12—C13—C14	121.8 (2)	C17—C22—H22	119.00
C13—C14—C15	118.7 (2)	C21—C22—H22	119.00
C10—C15—C14	121.2 (2)	O5—C23—H23A	109.00
N1—C16—C17	115.36 (19)	O5—C23—H23B	109.00
C16—C17—C18	120.0 (2)	O5—C23—H23C	109.00
C16—C17—C22	122.3 (2)	H23A—C23—H23B	110.00
C18—C17—C22	117.6 (2)	H23A—C23—H23C	110.00
C17—C18—C19	122.1 (2)	H23B—C23—H23C	109.00
C18—C19—C20	119.5 (2)		
			
C2—O2—C4—C5	2.3 (3)	O2—C4—C5—C6	176.2 (2)
C4—O2—C2—C1	−71.2 (3)	C5—C4—C9—C8	2.1 (4)
C4—O2—C2—C3	−166.47 (19)	O2—C4—C9—Cl2	3.1 (3)
C2—O2—C4—C9	−179.5 (2)	C9—C4—C5—C6	−1.9 (4)
C23—O5—C20—C21	−180.0 (2)	C4—C5—C6—C7	0.1 (4)
C23—O5—C20—C19	−1.0 (4)	C5—C6—C7—Cl1	−177.73 (19)
C16—N1—C1—C2	−173.9 (2)	C5—C6—C7—C8	1.4 (4)
C3—N1—C1—O1	−177.6 (3)	C6—C7—C8—C9	−1.2 (4)
C1—N1—C16—C17	103.0 (3)	Cl1—C7—C8—C9	177.94 (18)
C3—N1—C16—C17	−72.5 (3)	C7—C8—C9—Cl2	−179.75 (19)
C16—N1—C3—C10	−70.0 (3)	C7—C8—C9—C4	−0.5 (4)
C1—N1—C3—C2	−2.85 (19)	C3—C10—C15—C14	−177.0 (2)
C16—N1—C3—C2	173.8 (2)	C3—C10—C11—C12	175.9 (2)
C3—N1—C1—C2	2.90 (19)	C11—C10—C15—C14	−0.1 (3)
C1—N1—C3—C10	113.4 (2)	C15—C10—C11—C12	−1.1 (4)
C16—N1—C1—O1	5.7 (5)	C10—C11—C12—C13	1.1 (4)
O4—N2—C13—C14	−0.5 (4)	C11—C12—C13—N2	−179.8 (2)
O3—N2—C13—C12	−1.0 (4)	C11—C12—C13—C14	0.0 (4)
O4—N2—C13—C12	179.2 (3)	N2—C13—C14—C15	178.7 (2)
O3—N2—C13—C14	179.2 (3)	C12—C13—C14—C15	−1.1 (4)
N1—C1—C2—C3	−2.72 (18)	C13—C14—C15—C10	1.2 (4)
O1—C1—C2—O2	67.4 (4)	N1—C16—C17—C22	−47.4 (3)
O1—C1—C2—C3	177.8 (3)	N1—C16—C17—C18	136.2 (2)
N1—C1—C2—O2	−113.1 (2)	C16—C17—C22—C21	−176.6 (2)
C1—C2—C3—N1	2.49 (16)	C18—C17—C22—C21	−0.1 (4)
O2—C2—C3—C10	2.4 (3)	C16—C17—C18—C19	175.8 (2)
C1—C2—C3—C10	−115.4 (2)	C22—C17—C18—C19	−0.8 (4)
O2—C2—C3—N1	120.25 (19)	C17—C18—C19—C20	1.3 (4)
C2—C3—C10—C15	94.1 (3)	C18—C19—C20—C21	−0.9 (4)
N1—C3—C10—C15	−4.9 (3)	C18—C19—C20—O5	−179.9 (2)
C2—C3—C10—C11	−82.7 (3)	O5—C20—C21—C22	179.1 (2)
N1—C3—C10—C11	178.3 (2)	C19—C20—C21—C22	0.0 (4)
C5—C4—C9—Cl2	−178.70 (19)	C20—C21—C22—C17	0.5 (4)
O2—C4—C9—C8	−176.2 (2)		

### Hydrogen-bond geometry (Å, º)

Cg is the centroid of the C17–C22 benzene ring.

**Table:**

*D*—H···*A*	*D*—H	H···*A*	*D*···*A*	*D*—H···*A*
C3—H3···O1^i^	0.98	2.58	3.417 (3)	143
C6—H6···O5^ii^	0.93	2.57	3.328 (3)	139
C12—H12···O3^iii^	0.93	2.57	3.495 (4)	176
C16—H16*A*···*Cg*^iv^	0.97	2.70	3.649 (3)	166

Symmetry codes: (i) *x*−1, *y*, *z*; (ii) −*x*, −*y*, −*z*; (iii) −*x*−1, −*y*+1, −*z*; (iv) *x*+1, *y*, *z*.

## References

[bb1] Akkurt, M., Türktekin, S., Jarrahpour, A., Badrabady, S. A. T. & Büyükgüngör, O. (2011). *Acta Cryst.* E**67**, o183. 10.1107/S1600536810052645 PMC3050394 21522688

[bb2] Arya, N., Jagdale, A. Y., Patil, T. A., Yeramwar, S. S., Holikatti, S. S., Dwivedi, J., Shishoo, Ch. J. & Jain, K. S. (2014). *Eur. J. Med. Chem.* **74**, 619–656. 10.1016/j.ejmech.2014.01.002 24531200

[bb3] Banik, I., Becker, F. F. & Banik, B. K. (2003). *J. Med. Chem.* **46**, 12–15. 10.1021/jm0255825 12502355

[bb4] Butcher, R. J., Akkurt, M., Jarrahpour, A. & Badrabady, S. A. T. (2011). *Acta Cryst.* E**67**, o1101–o1102. 10.1107/S1600536811013018 PMC3089293 21754420

[bb5] Delpiccolo, C. M. L., Fraga, M. A. & Mata, E. G. (2003). *J. Combin. Chem.* **5**, 208–210. 10.1021/cc020107d 12739933

[bb6] Farrugia, L. J. (2012). *J. Appl. Cryst.* **45**, 849–854.

[bb7] France, S., Weatherwax, A., Taggi, A. & Lectka, T. (2004). *Acc. Chem. Res.* **37**, 592–600. 10.1021/ar030055g 15311958

[bb8] Hodous, B. L. & Fu, G. C. (2002). *J. Am. Chem. Soc.* **124**, 1578–1579. 10.1021/ja012427r 11853423

[bb9] Pitts, C. R. & Lectka, T. (2014). *Chem. Rev.* (Epub ahead of print). PMID: 24555548 [PubMed - as supplied by publisher] 10.1021/cr4005549 24555548

[bb10] Schunk, S. & Enders, D. (2000). *Org. Lett.* **2**, 907–910. 10.1021/ol0055465 10768183

[bb11] Sheldrick, G. M. (2008). *Acta Cryst.* A**64**, 112–122. 10.1107/S0108767307043930 18156677

[bb12] Stoe & Cie (2002). *X-AREA* and *X-RED32* Stoe & Cie, Darmstadt, Germany.

